# Review of policy and legislative framework for disability services in Namibia

**DOI:** 10.4102/sajp.v74i1.399

**Published:** 2018-03-27

**Authors:** Tonderai W. Shumba, Indres Moodley

**Affiliations:** 1 School of Nursing and Public Health, University of KwaZulu-Natal, South Africa

## Abstract

**Background:**

The Namibian policies and legislative framework were reviewed to determine the extent to which the needs of persons with disabilities were met and aligned with the United Nations Convention on the Rights of Persons with Disabilities (UNCRPD). Further, the disability legislative framework of Namibia is compared with that of other southern African countries.

**Methods:**

We conducted a retrospective analysis of policy and legal framework which addresses the needs and rights of persons with disabilities in Namibia from 1990 to 2016. A qualitative approach employing a case study design was used. Furthermore, a comparative analysis of the policies and legislation for alignment with the UNCRPD and how Namibia compares with other southern African countries is discussed.

**Results:**

Four policies, one piece of legislation and one international instrument were identified as directly related to disability. Community-based rehabilitation was adopted as the main strategy for rehabilitation. Alignment of the policy and legal framework with the UNCRPD was found to be minimal. Furthermore, most of the legislation in southern Africa was formulated before the existence of the UNCRPD in 2006.

**Conclusion:**

Although much progress has been made in meeting the needs of persons with disabilities, key implementation issues to be addressed include central coordination, overlapping strategies, disability models and gender differences. There is a need for the policy and legal framework of Namibia and other southern African countries to be more responsive to the human rights needs of persons with disabilities.

**Clinical implications:**

The study offers insights in reviewing disability policy and legal frameworks in southern Africa for influencing disability service delivery. Future studies can investigate the progress of implementation of disability policy and legal framework from the perspectives of implementers and recipients of services.

## Background

The United Nations (UN) General Assembly of 1993 (UN 1993) adopted resolution 48/96, the UN Standards Rules on the Equalisation of Opportunities for Persons with Disabilities (UNSREOP), that encompasses key areas including human rights, education, vocational training, access to the physical environment, transport and information for persons with disabilities. Although the UNSREOP resolution is non-binding, it served as a blueprint to inform member state policies and practices (Gesellschaft fur Technische Zusammenarbeit [GTZ] 2006). In Namibia, this blueprint formed the basis for formulation of the National Policy on Disability (NPD) (Government Republic of Namibia 1997). This NPD policy paved the way for other inclusive policies, legislation and programmes for persons with disabilities.

Along with the adoption of various disability policies, Namibia ratified the UN Convention on the Rights of Persons with Disabilities (UNCRPD) in 2007 (UN 2006). The UNCRPD and its Optional Protocol was adopted in 2006 and was opened for signature in 2007. It represents the first international comprehensive legally binding human rights treaty related to issues concerning persons with disabilities. Further, the UNCRPD is a key framework document on disability that guides all governments on the mechanisms for alignment with any new or existing policies, legislation and programmes. The ratification of the UNCRPD by Namibia confirms the commitment to uphold the human rights of persons with disabilities and a subsequent obligation to develop and review policies and legislation for ensuring alignment with the Convention.

Although Namibia has made much progress in establishing policies and legislation, there is no evidence of a study conducted to assess the policy and legislative framework and the extent to which this framework is aligned to the UNCRPD.

## Purpose

This review sets out to identify and assess the policy and legislative framework that addresses the needs of persons with disabilities in Namibia and the extent to which these are aligned (similarities and differences) with all articles of the UNCRPD. Further, the disability legislative framework of Namibia is compared with that of other southern African countries including Zimbabwe, Malawi and Zambia.

## Methods

We conducted a retrospective analysis of the policy and legal framework which addresses the needs and rights of persons with disabilities in Namibia from 1990 to 2016. A qualitative approach employing a case study design was used.

### Search strategy

All national policies or legislation developed post-independence from 1991 and any international conventions that were ratified by the national government were identified. The Namibian government websites were searched for relevant policies and legislation for disability-related subject matter, and manual searching within line ministries was undertaken. Only policies or legislation that directly addresses disability issues were included. The search was conducted in October 2016. Where hard copies of the policy or legislation were not available, reprints were requested from the line ministry responsible for overall coordination of the policy.

### Analytical framework

**FIGURE 1:**
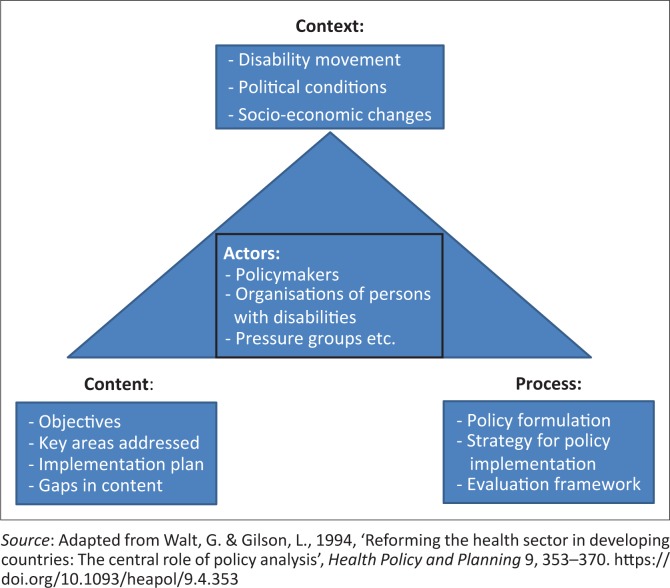
Policy triangle framework.

Review and analysis of the policy and legal framework was conducted in three steps. Firstly, the key contents of each document were thematically analysed utilising the Walt and Gilson (1994) policy triangle framework (Walt & Gilson 1994). This policy triangle framework enables identification of influencing contextual factors, the actors, the policy or Act contents and the processes with which the policy or Act was initiated, developed, implemented and evaluated (Figure 1).

Secondly, a comparative analysis was undertaken to determine alignment of the NPD and *National Disability Council (NDC) Act* with the UNCRPD (Article 1–50). This was done by extracting all the articles of the UNCRPD and adding them to the ‘data charting form’. Each Article in the UNCRPD was then mirrored with sections contained in both the NPD and *NDC Act* to determine the extent of alignment. A narrative summary of the results was completed.

Finally, a comparative analysis was conducted of the purposes and disability models adopted by the *NDC Act* with that of other disability legislation in previously mentioned southern African countries. The above analyses provided an overview and identified any gaps of interest in the disability policy and legal framework of Namibia.

### Ethical considerations

Ethical approval was obtained from the Human Sciences Research Ethics Committee at the University of KwaZulu-Natal (Ref No. HSS/0646/015D) and approval to collect data was obtained from the Research Committee at the Ministry of Health and Social Services in Namibia (Ref No. 17/3/3).

## Findings

The findings are presented in four parts. The first part outlines the progression of formulation and ratification of policies and legislative framework. The second part analyses the four components of policy: the context within which it was developed; the content and objectives; the actors involved in this policy; and the process of how the policy was initiated, formulated, implemented and evaluated (Figure 1). The third part presents the alignment of the key documents (NPD and *NDC Act*) to the UNCRPD. The fourth part reviews and compares the disability legislation in some identified southern African countries.

### Progression of formulation and ratification of policies and legislative framework

Prior to the development of any formalised approach to delivery of services for persons with disabilities in Namibia, a community-based rehabilitation (CBR) programme was adopted in 1992 which allowed steps to be taken to formalise and integrate the process for the provision of care and support for persons with disabilities within the primary health care network. In 1997, the NPD (Government Republic of Namibia 1997) was formulated, and this was followed by other policies and legislation, discussed below and summarised in Table 1.

**TABLE 1:** Progression of formulation and ratification of policies and legislative framework.

Policy or legislation	Year developed or ratified
National Policy on Disability	1997
National Policy on Orthopaedic Technical Services	2001
*National Disability Council Act*, 2004 (Act No. 26 of 2004)	2004
National Policy for Mental Health	2005
United Nations Conventions of Persons with Disabilities	2007 (ratification)
Sector Policy on Inclusive Education	2013

### Context, content, actors and process in the development of policy and legal framework in Namibia

A summary of the context, content, actors and process in the development policy and legal framework in Namibia is displayed in Table 2 and summarised subsequently.

**TABLE 2:** Context, content, actors and process in the development of policy and legal framework in Namibia.

Policy or legislation	Context	Content	Actors	Process
National Policy on Disability (1997)	- Need to create a ‘Society for All’ based on the principles of the Standard Rules on the Equalisation of Opportunities for Persons with Disabilities - to achieve a full social integration of persons with disabilities	- CBR adopted as the main strategy for rehabilitation - Special target groups are: women, children and elderly with disabilities particularly in rural areas - Four cornerstones on which key concern areas were built. However they are broad and ambiguous - Guided by the principles of the Standard Rules on the Equalisation of Opportunities for Persons with Disabilities - Adopt a medical and social model	- Coordinators: Ministry of Lands (1997–2005), Ministry of Health and Social Services (2005–2015), Department of Disability Affairs, Office of Vice President (2015 to date) - Stakeholders: All line ministries, persons with disabilities	- The principles of the Standard Rules on the Equalisation of Opportunities for Persons with Disabilities served as leading guidelines and also formed the basis for implementation - The policy represented Article 2 of ILO Convention No. 159 on the Vocational Rehabilitation and Employment of Disabled Persons - Literature review but no evidence of use of operational research in formulation - No evidence of extensive stakeholder consultation in development
National Policy on Orthopaedic Technical Services (2001)	- To overcome the physical and social barriers faced by persons with physical disabilities when it comes to accessibility to public and private facilities - The need for orthoses and prostheses was approximately 0.5% of the population which comprised 8500 persons	- No monitoring and evaluation framework proposed leads to lack of accountability - Used the WHO classifications of impairments, disability and handicap (WHO-A29/INF.Doc/1,1996) and that is using a medical model of disability - Policy principles guided by the Standard Rules on the Equalisation of Opportunities for Persons with Disabilities and the National Policy on Disability - Implementation plan in place - CBR facilitates identification, screening and referral for orthopaedic services	- Coordinator: Ministry of Health and Social Services - Stakeholders: Ministry of Education, Ministry of Labour and Social Welfare, Ministry of Works, Transport and Communication, persons with disabilities	- Use of evidence in formulation (rapid assessments, operational research) - The 1991 Cabinet approval of a working document on integration of persons with disabilities initiated process - In 1994 Ministry of Health and Social Services realised the need to reach rural population - The National Development Plan 1 (NDP1 1995/1996–1999/2000) was initiated and formed establishment of nationwide Orthopaedic Technical Services - Development guided by the concepts of the Standard Rules on the Equalisation of Opportunities for Persons with Disabilities and the National Policy on Disability - Stakeholder consultation was limited to targeted concerned parties - Key indicators set that are supposed to be annually reviewed through work plans
*National Disability Council Act*, 2004 (Act No. 26 of 2004)	- Need to establish a National Disability Council as a monitoring body	- To provide for the functions, powers and composition of National Disability Council - National Disability Council may gather information, disseminate information and raise awareness regarding persons with disabilities - Line ministries to report annually, no monitoring and evaluation framework proposed leads to lack of accountability - No regulations in place	- Coordinator: National Disability Council - Stakeholders: All line ministries, Organisations of Persons with Disabilities, persons with disabilities	- National Disability Council to monitor implementation of the National Policy on Disability - All line ministries to report annually to the National Disability Council and the minister responsible for rehabilitation then reports to cabinet
National Policy on Mental Health (2005)	- Need for the extension of mental health services to communities - The need to protect the rights of people with mental disorders - Lack of evidence-based mental health services	- Guided by national and international legal frameworks - Outlines strategies and institutional framework for implementation - Proposed formulation of a strategic plan and guidelines to enhance implementation. - Policy targets set but no monitoring and evaluation framework proposed leads to lack of accountability - Adopts both a medical and human rights model of disability - CBR facilitates identification, screening and referral for mental health services	- Coordinator: Ministry of Health and Social Services - Stakeholders: Mental Health Action Group (representatives from line ministries), University of Namibia, representatives of non-governmental organisations, faith-based organisations, private sector and communities	Internal reviews of activities are done annually - Use of evidence in formulation (rapid assessments, benchmark tours) but lacks operational research
Sector Policy on Inclusive Education (2013)	- Need to pave the way for all children in Namibia to learn and participate fully in the education system particularly in ‘mainstream schools’ - Need to educate learners in least-restrictive environments near their neighbourhood	- Outlines strategies and their specific outcomes - Monitoring and evaluation framework proposed leads to accountability - Implementation plan proposed leads to accountability - CBR facilitates identification, screening and referral for education	- Coordinator: Ministry of Education - Stakeholders: Inter-ministerial committee (consists of senior government officials), NGOs, Regional Councils, University of Namibia	- Guided by national and international legal frameworks - Extensive literature review and stakeholder consultation in development leads to clear objectives - Key indicators set that are supposed to be annually reviewed through work plans

*Source*: Adapted from Walt, G. & Gilson, L., 1994, ‘Reforming the health sector in developing countries: The central role of policy analysis’, *Health Policy and Planning* 9, 353–370. https://doi.org/10.1093/heapol/9.4.353

CBR, community-based rehabilitation; NGOs, non-governmental organisations.

#### Context

Findings revealed that the development of the policy and legal framework in Namibia was triggered by the need to address the barriers faced by persons with disabilities. The NPD (Government Republic of Namibia 1997) has an overall aim of creating a ‘Society for All’. On the contrary, The National Policy on Orthopaedic Technical Services (Ministry of Health and Social Services [MoHSS] 2001) addresses access to assistive devices, whereas the National Policy for Mental Health (MoHSS 2005) promotes mental health services and protection of rights of persons with mental health issues. Although the Sector Policy on Inclusive Education (Government Republic of Namibia 2013) places a strong emphasis on children with disabilities, it also addresses the needs of other children and young people who are educationally marginalised. Of the four policies, the National Policy for Mental Health is the only one that explicitly addresses human rights issues.

The *NDC Act* (Government Republic of Namibia 2004) was enacted specifically to establish the NDC with a mandate to identify issues that need to be addressed concerning persons with disabilities. Further, the NDC has the mandate to monitor the implementation of the NPD.

#### Content

The NPD stipulates that CBR is the main strategy to drive the implementation for disability and rehabilitation services in the country. The National Policy on Orthopaedic Technical Services, National Policy for Mental Health and Sector Policy on Inclusive Education use CBR services to assist CBR in early identification and screening.

Notably, all the policies have an implementation plan that potentially assists with easy execution. Notwithstanding the value of monitoring and evaluation frameworks, all policies with the exception of the Sector Policy on Inclusive Education have no monitoring and evaluation framework.

The implementation of disability policies and legal framework is also guided by the model of disability adopted. All disability policies identified adopted different models of disability. The NPD and the Sector Policy on Inclusive Education adopt a medical and social model, whereas the National Policy on Orthopaedic Technical Services adopts a medical model and the National Policy for Mental Health adopts both a medical and human rights model of disability. Further, the *NDC Act* adopts both the medical and social model on disability. On the premise that Namibia ratified the UNCRPD, there is a need to review and standardise the policy and legal framework in order to embrace the social and human rights model adopted by the Convention.

Notably, the main purpose of the *NDC Act* is to provide for the functions, powers and composition of the NDC. Further, it stipulates that all line ministries are to report annually to the NDC on their progress in mainstreaming persons with disabilities in various services and programmes.

#### Actors

The implementation of policies is fragmented and resides in various line ministries. The NDC has the mandate for coordinating the NPD. However, the coordination of both the National Policy on Orthopaedic Technical Services and the National Policy for Mental Health falls under the MoHSS and that of the Sector Policy on Inclusive Education under the Ministry of Education.

Although the mandate of coordinating individual policies is with a specific government ministry, the mandate for coordinating the activities of the NDC rests with the minister responsible for rehabilitation. No evidence identifying which entity is responsible for ensuring compliance with the UNCRPD could be found. Further, there is no formal overarching coordination of the policies and programmes to ensure seamless implementation.

Both the *NDC Act* and the UNCRPD place the responsibility for legislation promulgation and policy implementation on all government line ministries. The NPD places the responsibility for policy implementation on all line ministries, whereas the National Policy on Orthopaedic Technical Services (MoHSS 2001) shares responsibility with several government line ministries including Ministries of Education, Labour, Works, Transport and Communication and Community. Both the National Policy on Mental Health (MoHSS 2005) and the Sector Policy on Inclusive Education (Government Republic of Namibia 2013) have ministerial coordinating committees.

#### Processes

Although policies have been in place for over 10 years (except the Sector Policy on Inclusive Education), none has been reviewed or updated. Only the Sector Policy on Inclusive Education has a clearly laid out monitoring and evaluation framework that stipulates the outcomes, implementers, budget, time frames and recommendations. The other policies (NPD, National Policy on Orthopaedic Technical Services and National Policy for Mental Health) simply suggested annual internal reviews. It is noteworthy that the mandate for reviewing the policies is placed on the coordinating ministry except the NPD where the mandate is on the NDC.

The *NDC Act* has no monitoring and evaluation framework in place to guide the process on amending or reviewing the Act. Furthermore, the *NDC Act* has no regulations that guide its implementation as per Section 23. Contrary to the *NDC Act*, the UNCRPD provides a monitoring framework at national and international levels. Article 33 and Article 34 of the UNCRPD provide mechanisms of national and international monitoring, respectively.

It is critical for policy formulation to be underpinned by the use of evidence in formulation (operational research, literature reviews, rapid assessments, benchmark tours). The National Policy on Orthopaedic Technical Services, the National Policy on Mental Health and the Sector Policy on Inclusive Education indicate the use of extensive evidence during formulation, whereas the NPD and *NDC Act* did not show any evidence of extensive use of evidence in their formulation.

### Alignment of the National Policy on Disability and *National Disability Council Act* with the United Nations Convention on the Rights of Persons with Disabilities

The policy and legal framework in Namibia predates the UNCRPD. It was therefore critical to investigate the extent of alignment of the NPD and *NDC Act* with all of the articles of the UNCRPD. All the articles of the UNCRPD were reviewed and compared for alignment with sections of both the NPD and *NDC Act* as shown in Table 3.

**TABLE 3:** Comparison of United Nations Convention on the Rights of Persons with Disabilities (Article 1–50) with National Policy on Disability and *National Disability Council Act.*

UNCRPD articles	Alignment of *NDC Act* Sections to UNCRPD Articles	Alignment of NPD Sections to UNCRPD Articles
1. Purpose: purpose and definition of disability	Section 1	Section 1.1
2. Definitions: definition of key terms	Section 1	Section 1.1, 1.2
3. General principles: respect for dignity and individual autonomy, non-discrimination, full and effective participation, respect for difference and acceptance, equality of opportunity, accessibility, equality between men and women, respect for the evolving capacities of children with disabilities	Not aligned	Section 2.4
4. General obligations: promoting conforming legislation, eliminating discrimination, promoting research, best practices, training; providing accessible information	Not aligned	Section 3.1.2;3.2.4;3.1.5;3.2.16; 3.2.17;3.2.18
5. Equality and non-discrimination: affirm equality, prohibit discrimination	Section 22	Section 2.1; 3.1.2; 3.2.5; 3.2.16; 3.2.17; 3.2.17;3.2.19
6. Disabled women: advance and empower women	Not aligned	Section 2.5.1; 3.2.19
7. Disabled children: ensure that children with disabilities experience human rights and fundamental freedoms	Not aligned	Section 2.5.2
8. Awareness raising: promote awareness of disabilities, dispel stereotypes, highlight contributions of people with disabilities	Section 16	Section3.2.1
9. Accessibility: promote independent living and full participation, accessible environment	Not aligned	Section 3.2.4
10. Right to life: affirm right to life for all persons, including people with disabilities	Not aligned	Not aligned
11. Risk and humanitarian emergencies: ensure protection and safety in armed conflict, humanitarian emergencies, natural disasters	Not aligned	Not aligned
12. Equal recognition before the law: support exercise of legal capacity on an equal basis with others	Section 15; 22	Section3.2.16
13. Equal access to justice: provide for access to justice – reasonable accommodations, training in justice system	Not aligned	Section 3.2.16
14. Liberty and security of person: not justify deprivation of liberty solely on basis of disability	Not aligned	Not aligned
15. Freedom from torture: protect from medical or scientific experimentation without consent and from inhuman or degrading treatment or punishment	Not aligned	Not aligned
16. Freedom from exploitation: protect within and outside home, provide gender and age-sensitive assistance, monitor disability programmes	Not aligned	Section 3.2.19
17. Protecting the integrity of the person: respect physical and mental integrity.	Not aligned	Section 3.2.19
18. Liberty of movement and nationality: recognise right to choose residence, change nationality, have right to identification or to travel, register children after birth, ensure right to a name and nationality and parental care	Not aligned	Not aligned
19. Independent living and community participation: recognise right to community living, choices in their place of residence, access to a range of support services, and equality of access to community services and facilities	Not aligned	Section 3.1.1; 3.2.2
20. Personal mobility: promote access to mobility services and supports; provide training in mobility skills	Not aligned	Not aligned
21. Freedom of expression and opinion; access to information: ensure communication in form of their choice	Not aligned	Section 3.2.5; 3.2.10
22. Respect for privacy: protect the privacy of personal communications and health and rehabilitation information	Not aligned	Not aligned
23. Respect for home and family: eliminate discrimination in marriage, family, parenthood, and relationships; ensure non-discrimination in fertility	Not aligned	Section 3.2.19
24. Education: promote inclusive and equal education; provide support for academic and social development	Not aligned	Section 3.2.6; 3.2.7
25. Health: promote access to gender-sensitive health, reproductive services in community and on non-discriminatory basis; prevent denial of health care and food or liquids on the basis of disability	Not aligned	Section 3.1.1; 3.2.2;3.2.3
26. Habilitation and rehabilitation: provide community and strengthen need-based services	Not aligned	Section 3.1.1; 3.2.3
27. Work and employment: prohibit discrimination, promote safe and equal working conditions and equal work for equal value; ensure reasonable accommodations; develop employment supports	Not aligned	Section 3.2.8; 3.2.9
28. Adequate standard of living and social protection: ensure right to adequate food, clean water, clothing, housing and social programmes	Not aligned	Section 3.2.13; 3.2.15
29. Participation in political and public life: ensure right to vote or be elected, accessible voting procedures and materials, free expression	Not aligned	Not aligned
30. Participation in cultural life: ensure accessible cultural materials and locations, encourage participation in mainstream sporting activities, ensure access to activities in the school system	Not aligned	Section 3.2.11; 3.2.12
31. Statistics and data collection	Section 15	Section 3.2.18
32 to 50 – responsibilities of countries that ratified on implementing, monitoring and reporting on the UNCRPD on its effect	UN reporting system (Both documents developed before UNCRPD, thus no alignment)	

UNCRPD, United Nations Convention on the Rights of Persons with Disabilities; NDC, National Disability Council; NPD, National Policy on Disability.

The findings revealed that there is some alignment of the UNCRPD Articles with sections of the NPD with the exception of UNCRPD Articles 10 (Right to life), 11 (Risk and humanitarian emergencies), 14 (Liberty and security of person), 15 (Freedom from torture), 18 (Liberty of movement and nationality), 20 (Personal mobility), 22 (Freedom of expression and opinion; access to information), which are not reflected in either the NPD or the *NDC Act*. Articles 32 to 52 of the UNCRPD stipulate the UN reporting system. Both the NPD and *NDC Act* were developed before UNCRPD and thus do not show any alignment.

Minimal alignment is shown between the UNCRPD Articles and the sections in the *NDC Act*. Alignment of the *NDC Act* is only apparent in Articles 1 (Purpose), 2 (Definitions), 5 (Equality and non-discrimination), 6 (Disabled women), 8 (Awareness raising), 12 (Equal recognition before the law) and 31 (Statistics and data collection).

Identification of the gaps in alignment of policy and legislation with the UNCRPD can provide an opportunity for areas of amendment. The UNCRPD is the foundation upon which Namibia needs to build and revise its national policies and laws. This will further facilitate disability being addressed as a human rights issue and thus stimulate all sectors in Namibia to mainstream disability into their activities.

### Comparison of the *National Disability Council Act* and those in selected southern African countries

Despite the misalignment of the *NDC Act* and the UNCRPD as shown in this review, the Act has good elements that can be compared with other southern African countries. Findings indicated that except for Malawi, most of the Acts in southern Africa were formulated before the coming into existence of the UNCRPD in 2006. With the exception of Zambia, most of the Acts have not been amended to align them to the UNCRPD. The main purpose of the *NDC Act of Namibia* (2004) was to establish a monitoring body for the implementation of disability services. This purpose is similar to that of *Disabled Persons Act* (Zimbabwe) and *Persons with Disabilities Act* (Zambia). The *NDC Act of Namibia* does not make specific provision for the promotion and protection of human rights which the *Disability Act of Malawi* does. The definitions adopted by the *NDC Act of Namibia* are similar to those of the *Disabled Persons Act* (Zimbabwe) and *Disability Act of Malawi*.

## Discussion

This review identified that CBR underpins all policies and legislation as a key strategy for providing rehabilitation services in Namibia. Thus, the CBR programme plays a central role in the implementation process of disability services in Namibia.

Key to the development of any policy is the ‘actor power’ which is explained by Shiffman and Smith (2007) as the individual or organisational strength related to the issue at hand. Although the policy and legal framework explained the main actors and their influence, it varied in the descriptions and degree of involvement as key grassroots actors, including the involvement of persons with disabilities. The descriptions were strong with National Policy on Orthopaedic Services, National Policy for Mental Health and Sector Policy on Inclusive Education and weak with the NPD and *NDC Act*. Policy formulation should take into account the refrain by persons with disabilities that is ‘nothing about us without us’. Similarly, under the aegis of Article 32 of the UNCRPD, persons with disabilities should be consulted on services in which they are involved (UN 2006).

One of the key issues identified in the review was coordination. It appears that all the policies and legislation pertaining to disability are coordinated separately, often by different government ministries or entities as is the case with NDC. The potential for confusion and duplication is created when there is lack of a central mechanism for policy coordination leading to implementers being required to report to several coordinators from different departments (Percy 1993). Further, the change in the coordination function of the NPD in Namibia from one ministry to the other has not allowed for continuity of implementation and has encountered or endured changes in approach depending on disability models being imposed. For example, coordination functions from the Ministry of Lands, Resettlement and Rehabilitation with a focus on the social model of disability were moved to the MoHSS with a focus on the medical model. Similarly, the *NDC Act* stipulates that the minister responsible for rehabilitation coordinates its implementation. The minister responsible for rehabilitation has been changing, and often these changes in coordination with functions can hinder effective or efficient implementation.

Within sections of the policies reviewed, there are multiple strategies and this creates a multiplicity of tasks and responsibilities by a number of implementing ministries. For example, the NPD presents 22 strategies to be implemented by all ministries. The high number of role players involved in the NPD can create multiple decision points that may be in conflict with each other. This can cause inertia in implementation and policy analysis when coordination is spread over many actors and resources are spread over multiple competing actors with opposing directives (Percy 1993). There are some conceptual challenges that can emerge during policy analysis including capturing and measuring levels of resources and power of diverse actors (Walt et al. 2008). Thus, analysis of the NPD becomes inaccurate as the ministries have continuously been changed with various changing governments.

Findings from this study revealed that the policy and legal framework in most instances was not guided by adequate use of evidence (rapid assessments, benchmark tours, literature reviews, operational research) in formulation. This may have been attributed to the demand for quick answers and remedies by policymakers which can lead to reductionism (Walt et al. 2008). To this end, a lack of evidence can lead to ambiguous objectives and strategies that can impede implementation, as it takes time for implementers to understand the policy. For example, the NPD and the National Policy for Mental Health give responsibility to all ministries, but does not explicitly spell out their tasks and powers. In contrast, the Sector Policy on Inclusive Education is the only policy reviewed that has clear objectives and strategies for the implementers.

A lack of guidelines was apparent in most policies with the *NDC Act* not having regulations to guide implementation. Guidelines are critical in promoting action (Walt & Gilson 2014). Similarly, the UNCRPD has not been cascaded to the national level for easier implementation. Thus, the lack of guidelines for application of the UNCRPD to national policies and legislation poses a challenge to effective implementation.

Another crucial issue that was not addressed adequately by the policies and legal framework is gender differences. Although the NPD mentions women as a special focus group, it does not pay sufficient attention to gender differences that should be addressed such as the role of women as a wife or mother. The role of women and men in the house can ultimately affect their career paths. Men with disabilities are expected to have a caregiver who does household chores, yet a woman with a disability is expected to do all this by herself (Percy 1993). Therefore, this male-centred bias in policy and legal formulation hampers the efforts of embracing the human rights model of disability stipulated in the UNCRPD.

The policy and legal framework adopted varying models of disability and definitions. This might have been because of the different stages when the policies and legislation were adopted as the disability models have evolved over time. Therefore, a lack of a uniform countrywide model on disability creates challenges with issues of eligibility for social protection, as there are no consistently applied assessments of disability. Furthermore, this inconsistency leads to difficulties in the provision of disability service delivery and evaluation.

Reflection on the alignment of the NPD and the *NDC Act* to the UNCRPD indicates a close alignment with some sections and minimal alignment with others. The minimal alignment of the *NDC Act* to the UNCRPD is because of the fact that the Act was enacted mainly to set up the National Council on Disability rather than regulating the implementation of the disability services. Moreover, the incompatibility of the NPD and the *NDC Act* to the UNCRPD may be attributed to the fact that both documents were formulated and pre-dated the UNCRPD. The disability framework in Namibia needs to be more responsive to the human rights of persons with disabilities, reasonably accommodate them and bring equality (Ntinda 2013). Similarly, most of the disability legislation in southern Africa predates the UNCRPD and thus does not align well with the human rights model of disability.

It is worth noting that Article 144 of the Constitution of Namibia (Government Republic of Namibia 1991) states that ‘international law and international agreements binding upon Namibia under this Constitution shall form part of the law of Namibia’ (Government Republic of Namibia 1991: 62). However, there is still potential value for developing countries to adopt specific policies and legislation to avoid a ‘one-size-fits-all’ approach that may not be congruent with the diversity of persons with disabilities in Africa. To this end, Namibia has made initiatives through the Office of the Ombudsman to develop a National Action Plan on Human Rights (Government Republic of Namibia 2014) which also addresses human rights issues for persons with disabilities. Similarly, the African Union through the African Commission on Human & Peoples’ Rights adopted a Draft Protocol to the African Charter on Human and Peoples’ Rights on the Rights of Persons with Disabilities in Africa. This protocol aims among others to mainstream disability in policies and legislation of member states (African Union 2016).

## Conclusion

Since independence in 1990, Namibia has made significant progress in developing policies and legislation to address the needs of persons with disabilities. This review identified the CBR programme as underpinning all policies and legislation as a key strategy for providing rehabilitation services in Namibia. The critical issues that could hamper the implementation process include among others the lack of a central mechanism for coordination, overlapping strategies, formulation not grounded in evidence, lack of regulations and guidelines, different disability models adopted and failure to address gender differences.

The NPD and the *NDC Act* indicate in certain instances a close and in others a minimal alignment to the UNCRPD. Further, most of the legislation and policies in southern Africa were formulated prior to the existence of the UNCRPD in 2006. The UNCRPD is the international gold standard instrument for promoting the rights of persons with disabilities. To this end, there is need for Namibia and other southern African countries to be more responsive to the human rights needs of persons with disabilities.

The finding of this study can be utilised to review the policy and legal framework of Namibia as well as offer insights into disability legislation in southern Africa. To strengthen the insights shared in this review, it is therefore important for future studies to investigate the progress of implementation of the policy and legal framework in Namibia from the point of view of the actual implementers and recipients of services. The diversity in disability groups potentially creates varying needs.
